# A dataset of distribution of antibiotic occurrence in solid environmental matrices in China

**DOI:** 10.1038/s41597-022-01384-5

**Published:** 2022-06-07

**Authors:** Qi Zhang, Guanshi Zhang, Dongsheng Liu, Xiu Zhang, Ruying Fang, Luqi Wang, Yunxiang Chen, Lingling Lin, Hongjuan Wu, Sen Li

**Affiliations:** 1 School of Environmental Science and Engineering, Huazhong University of Science and Technology, Wuhan, P. R. China; 2 Environmental Change Institute, University of Oxford, Oxford, UK

**Keywords:** Environmental impact, Risk factors, Environmental impact

## Abstract

While there is growing global concern about the impact of antibiotic residues on emergence and enhancement bacteria’s resistance, toxicity to natural organisms, and, ultimately, public health, a concise picture of measured environmental concentrations of antibiotic occurrence in multiple environmental matrices, particularly in solid matrices (e.g., sludge, soil, and sediments) is still elusive, especially for China. In this paper, we present an up-to-date dataset of the distribution of antibiotic occurrence in solid environmental matrices in China, derived from 210 peer-reviewed literature published between 2000 and 2020. We extracted geographical sampling locations and measured concentration associated with antibiotic occurrence reported in English and Chinese original publications, and applied quality-control procedures to remove duplicates and ensure accuracy. The dataset contains 6929 records of geo-referenced occurrences for 135 antibiotics distributed over 391 locations distinguished at four levels of scale i.e., provincial, prefectural, county, and township or finer. The geographical dataset provides an updated map of antibiotic occurrence in solid environmental matrices in China and can be used for further environmental health risk assessment.

## Abstract (summary)

**Table:** 

Measurement(s)	Scientific Publication
Technology Type(s)	digital curation
Factor Type(s)	location • matrices • antibiotics
Sample Characteristic - Environment	solid environmental material
Sample Characteristic - Location	China

## Background & Summary

Since the advent of penicillin in 1929, antibiotics have been widely used as effective disease prevention treatments and animal growth promoters^1,2^. Due to the low metabolic rate of humans and livestock, antibiotics are mainly excreted via urine and faeces^3,4^, and can migrate to effluent and sludge from domestic wastewater treatment plants (WWTPs), hospitals, and livestock farms either in their original form or in different metabolites^1,5^. A large fraction of these residual antibiotics has the potential to enter into solid environmental matrices through wastewater discharge, reclaimed water irrigation, and utilisation of animal manure and WWTP sludge as fertiliser in agriculture^4,6,7^. Contamination by residual antibiotics in municipal sludge^8–10^, soil^11–14^, and sediments^15–17^ has risen serious concerns, especially about antibiotic resistance, on its ultimate harm to public well-being and ecosystem health^12,18–20^. There is an urgent need to disclose the current pattern and environmental fate of antibiotics to better assess the risk of sewage discharge and future agricultural use of sludge products.

China has the world’s largest market of antibiotic products and consumes more than 25,000 tons antibiotics each year^21^. Several recent studies have reviewed and compiled the occurrence records of antibiotics in China, with foci on aquatic environment^21–25^, soil^26–30^, or WWTPs^31^. There is a lack of a comprehensive and systematic description of the geographic distribution of antibiotic occurrence in the solid environmental matrices in China, especially for municipal sludge, thus far, with Wang *et al*.^32^, Lyu *et al*.^2^, and Huang *et al*.^6^ being notable exceptions. Furthermore, as existing antibiotic-related datasets were on a coarse spatial resolution at the province-^2^ or basin-level^6,20^, a dataset with more detailed geographic information at finer scales would be promising for pinpointing regions at risk and modelling exercises towards environment and health management practices.

This paper presents an up-to-date dataset of the distribution of antibiotic occurrence in solid environmental matrices in China. The dataset described here comprises 6929 geo-referenced antibiotic occurrence records of 135 antibiotics in sludge, soil and sediments reported in 210 peer-reviewed publications from 2000 to 2020, covering 391 locations across China. The hotspots of antibiotic occurrence records are mainly located in the densely populated and economically prosperous regions of China, such as the Bohai Bay region, Beijing‒Tianjin‒Hebei region, Yangtze River Delta, and Pearl River Delta. Most studies focused on sediment and soil, while a limited number of studies (31 publications) have investigated municipal sludge. The top ten most frequently reported antibiotics are oxytetracycline, tetracycline, ciprofloxacin, sulfadiazine, sulfamethazine, norfloxacin, ofloxacin, sulfamethoxazole, roxithromycin, and enrofloxacin. The median values of antibiotic concentrations in the municipal sludge (15.30 μg/kg) are one to two orders of magnitude higher than that of soil (1.00 μg/kg) and sediments (0.40 μg/kg). In the future research, there is an essential need to strengthen surveillance of antibiotics over a broader geographical region, especially in southwest and northwest China.

## Methods

### Data collection

**Fig. 1:**
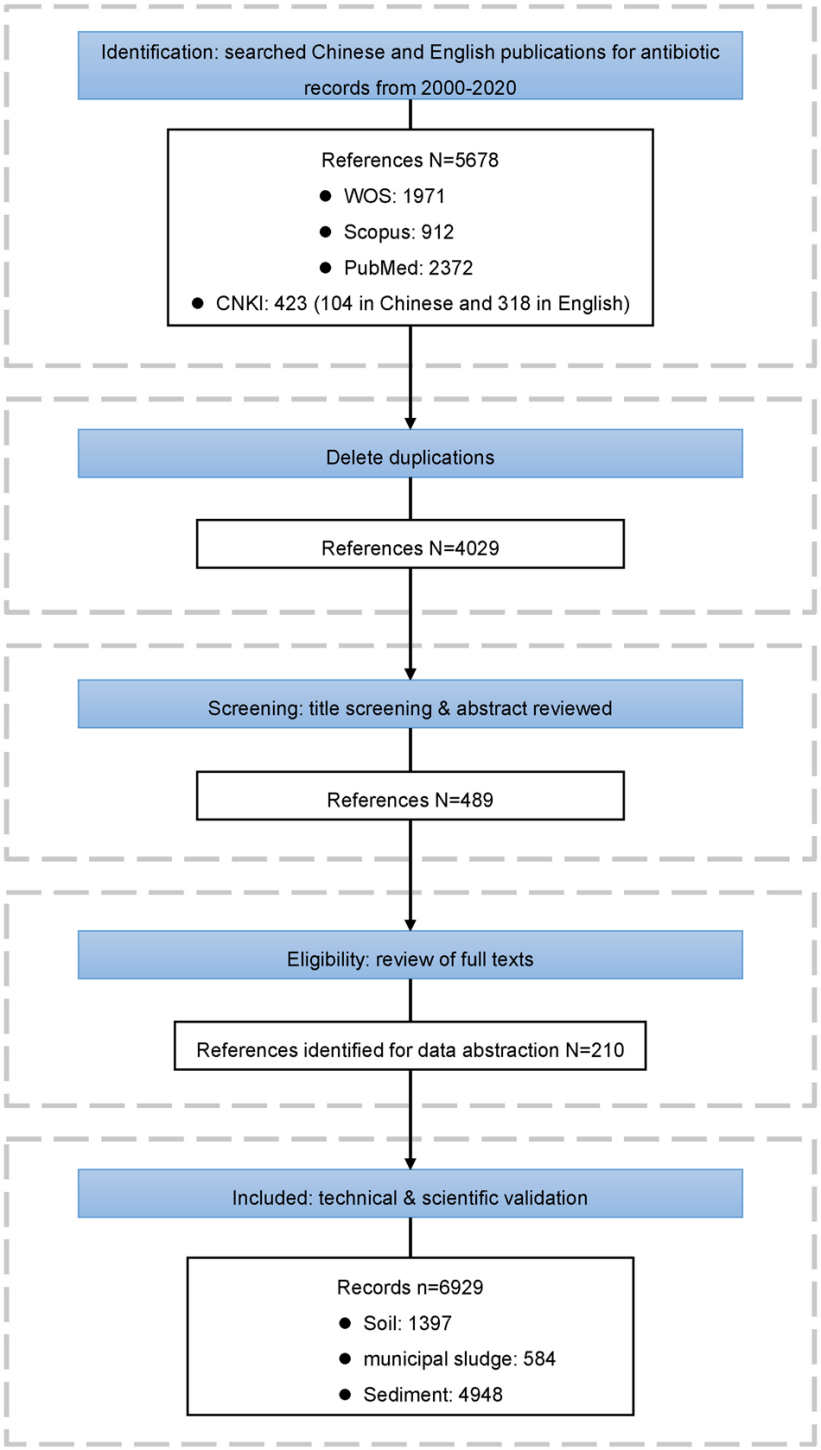
Schematic overview of the literature search procedure and results.

Our screening steps and selection criteria for literature review are outlined in Fig. 1. Publications (journal articles, conference proceedings, and degree theses, etc.) in both Chinese and English were collected by searching the four major scientific citation indexing services, the Web of Science (WOS) (https://www.webofscience.com/), the Scopus (https://www.scopus.com/), the PubMed (https://pubmed.ncbi.nlm.nih.gov/), and China National Knowledge Infrastructure (CNKI) (http://www.cnki.net/), respectively. A preliminary literature search disclosed that a significant number of antibiotic-related studies in China have been published after 2000. Therefore, we used the timeframe from January 2000 to November 2020. The keywords used for searching were (antibiotic* AND (soil OR sludge* OR biosolid* OR sediment*) AND China) with WOS, (antibiotic AND soil OR sludge OR biosolid OR sediment AND china) with Scopus, ((soil OR sludge OR biosolid OR sediment) AND antibiotic AND China) with PubMed, and (抗生素 * 中国 * (土壤 + 污泥 + 底泥 + 沉积物)) with CNKI. No language restrictions were placed on these searches.

**Fig. 2:**
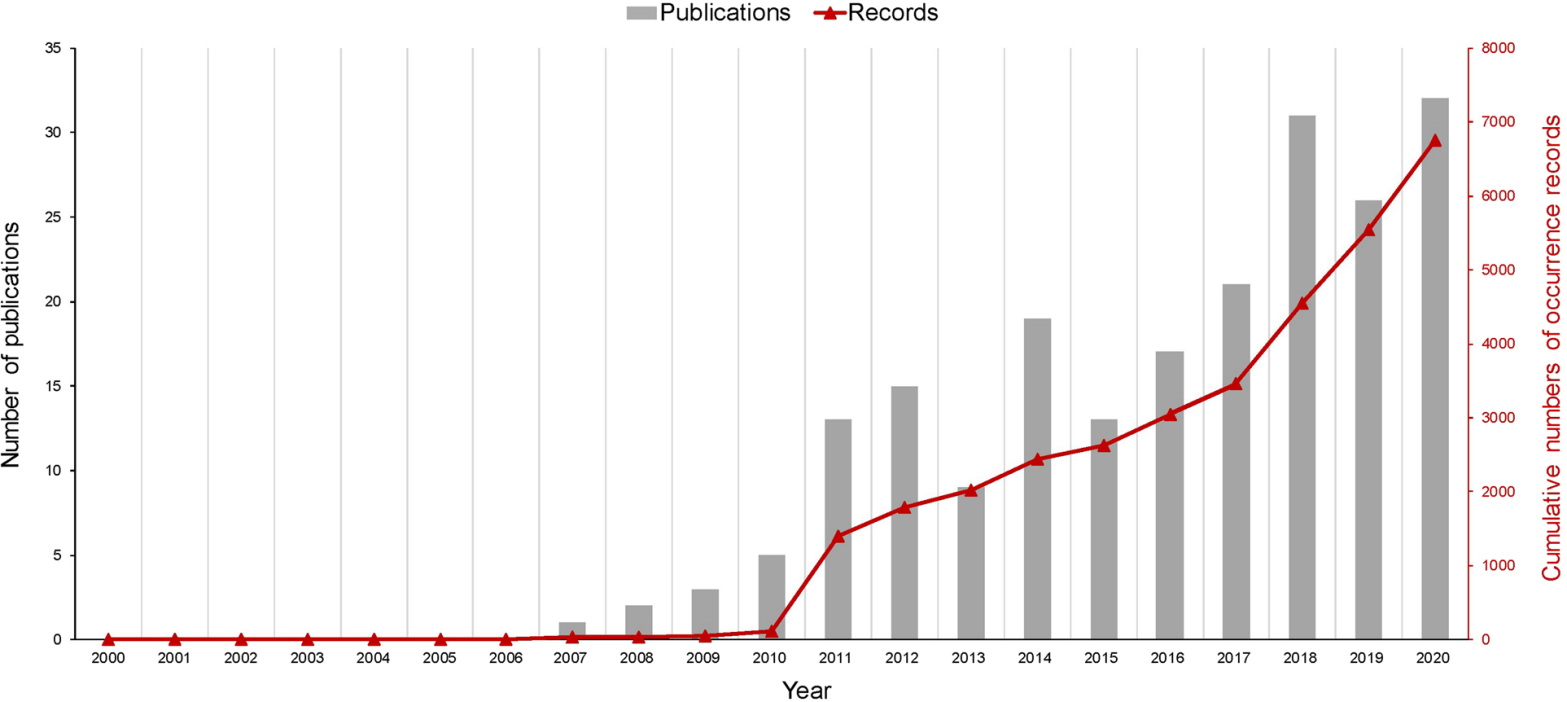
Increasing numbers of publications and records on the analysis of antibiotic occurrences in solid environmental matrices in China from 2000 to 2020.

A total of 5678 publications were compiled for screening, of which 1971 were retrieved from WOS, 912 from Scopus, 2372 from PubMed, and 423 from CNKI (104 in Chinese and 318 in English). We firstly checked through publications to eliminate duplicates. Secondly, abstracts of the remaining publications were examined and we excluded publications which only describe antibiotic resistant bacteria or antibiotic resistance genes not measured antibiotic concentration, or which only focus on human or organism not selected environment matrices. This resulted in 489 papers being selected for full-text review and further extraction of location and concentration information of antibiotic occurrence. Thirdly, having intensively read all the available full-texts, the publications which failed to report details of occurrence data and geographical information were further excluded, and finally, 210 publications were identified to be eligible for extraction. The very first study was published in 2007 in Water Research, and reported the concentration of ofloxacin, norfloxacin, roxithromycin, erythromycin-H_2_O, sulfadiazine, sulfadimidine, sulfamethoxazole, and chloramphenicol in municipal sludge at four sewage treatment plants in the Pearl River Delta^33^. The earliest article in Chinese was published in 2008, which reported five sulfonamides antibiotics in the soil in the Pearl River Delta^34^. In recent years, the number of publications of antibiotic occurrences in sludge, soil, and sediments has increased rapidly (Fig. 2). A full list of publications reviewed is provided in the online dataset^35^.

The key information extracted from the literature includes: (i) geographical location associated with antibiotic occurrence in sludge, soil, and sediments (and its geographic scale), (ii) information on the antibiotics examined (e.g., categories, chemical identifiers, etc.), (iii) concentration reported in standardized units (i.e., μg/kg or ng/g), (iv) sampling time, and (v) detection methods. It is common that an article reports several antibiotics at different locations and/or different time, and these records were separated so that each record in our dataset represents an occurrence of an antibiotic in a location reported in a specific time by an author. Concentrations that were reported as “not detected” or “below detection limit” were entered as “ND” ( = No Data, and regarded as zero value), using the similar approach adopted in a recent meta-analysis on the antibiotics found in global lakes^36^. The measured concentrations presented here were converted to the standardized units (μg/kg or ng/g). Four concentration values of each antibiotic were summarised in the dataset, including the maximum, minimum, mean, and median measured concentrations wherever available. It is worth noting that many studies on pharmaceutical residues in environmental matrices often target hotspots, resulting in measured concentrations outside of normal ranges. To facilitate potential users to distinguish these extreme values, the possible outliers in the concentration records were detected using the Tukey’s test^37^ and flagged in the dataset. Finally, 6929 records of occurrences of 135 antibiotics were compiled, of which 1397 records occurred in soil, 4948 in sediments, and 584 in municipal sludge. Antibiotics detected in solid environmental matrices were classified into the following categories: sulfonamides (30 chemicals), tetracyclines (22 chemicals), fluoroquinolones (28 chemicals), macrolides (17 chemicals), and β-lactams (13 chemicals) as well as other (25 chemicals). Detailed information on the antibiotics included in this study was listed in Supplementary Table 1.

### Geo-positioning

**Fig. 3:**
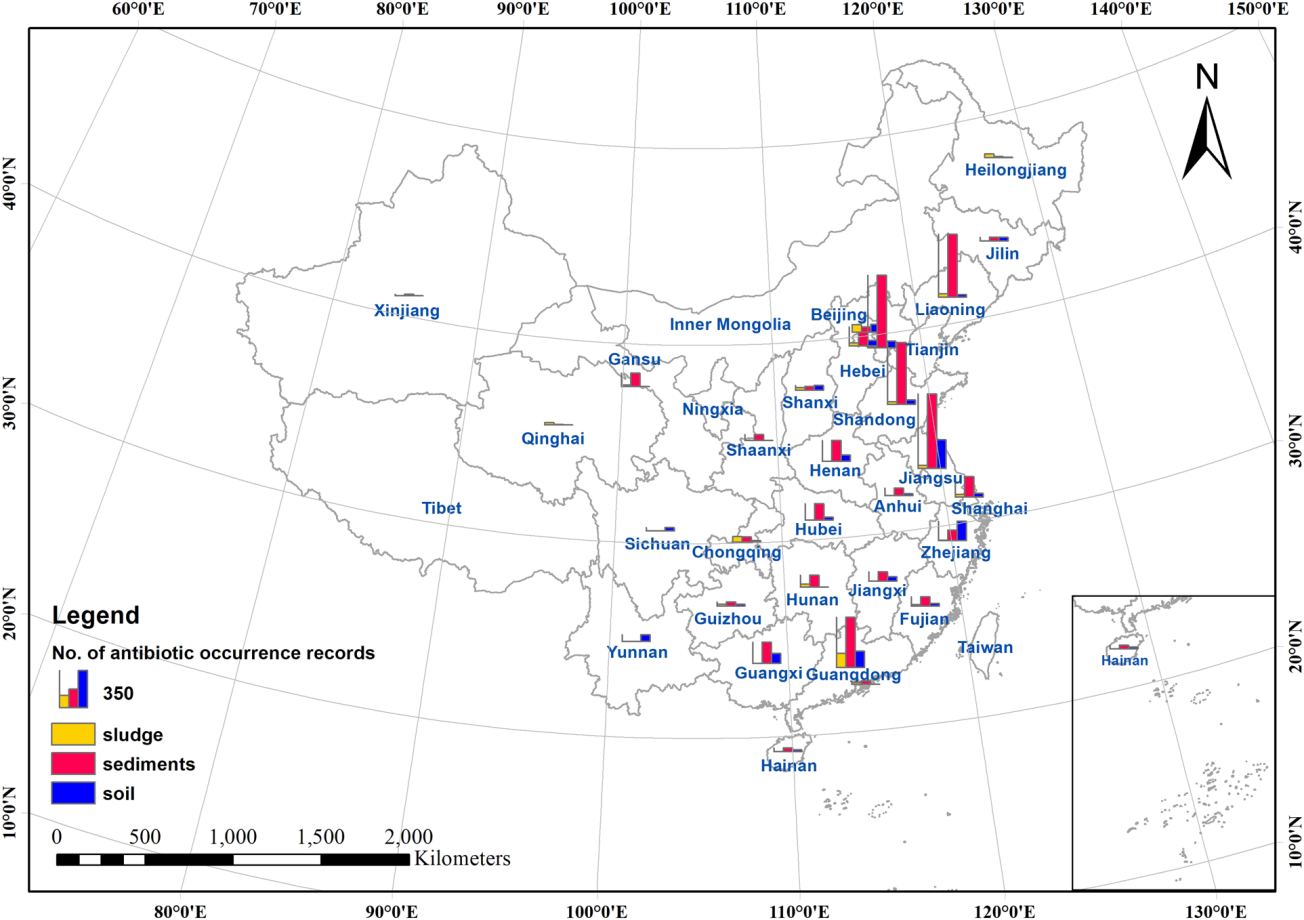
Number of antibiotic occurrence records in different solid environmental matrices by provincial-level divisions of China.

**Fig. 4:**
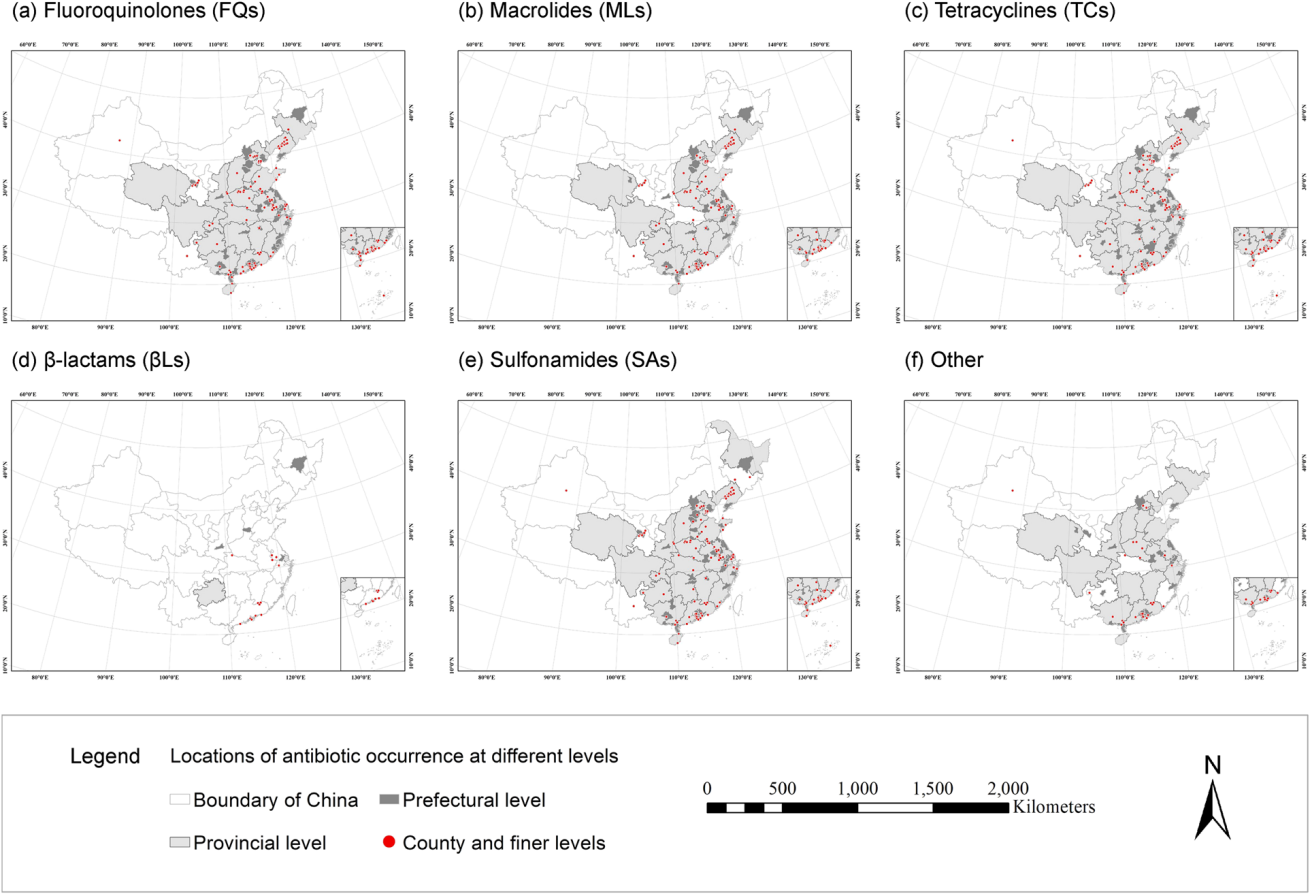
Locations of occurrence records of six antibiotic subcategories in solid environmental matrices (i.e. soil, sediments, and municipal sludge) in China. (**a**) Fluoroquinolones (FQs). (**b**) Macrolides (MLs). (**c**) Tetracyclines (TCs). (**d**) β-lactams (βLs). (**e**) Sulfonamides (SAs). (**f**) Other.

**Fig. 5:**
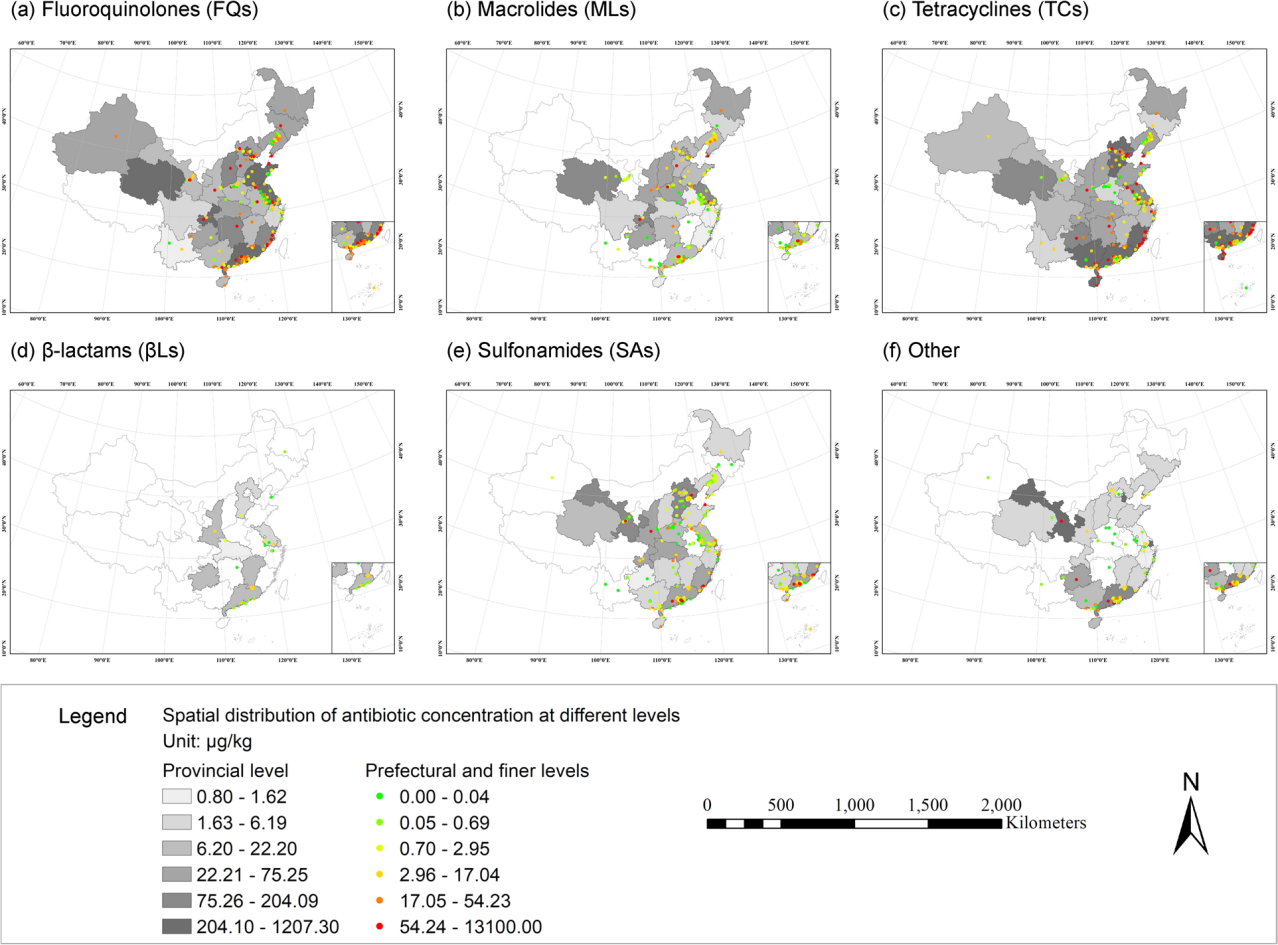
Spatial distribution of measured concentration of six antibiotic subcategories in solid environmental matrices (i.e. soil, sediments, and municipal sludge) in China. (**a**) Fluoroquinolones (FQs). (**b**) Macrolides (MLs). (**c**) Tetracyclines (TCs). (**d**) β-lactams (βLs). (**e**) Sulfonamides (SAs). (**f**) Other.

Unless the coordinates of sampling locations were provided, information on the geographical location needed to be extracted from the texts, tables, figures, and supplemental materials of the original publication. Following Zhang *et al*.^38^, we determined the latitudinal and longitudinal coordinates using Web APIs (Application Programming Interfaces) to access georeference functions of the most commonly used online location services in China, namely, Baidu Map (https://map.baidu.com/) and Amap (https://www.amap.com/). We searched keywords related to the location of each record, for example, the name of specific geographical objects, administrative regions, or water bodies, and recorded the latitude/longitude information. When only maps of the sampling sites were provided, we approximated rough coordinates through visual interpretation, mapped these records on Baidu Map or Amap, and then adjusted the coordinates according to the geographical characteristics of the original maps. In total number of 389 geographical locations were identified. Based on the level of geographical details, these locations were further classified into four different levels (i.e. provincial, prefectural, county, and township or finer level), which could help potential users of this dataset extract proper sections to use. Finally, the distribution of the reported antibiotic occurrence was visualized using ESRI ArcGIS 10.7 (Figs. 3, 4, 5). The administrative boundary map of China (2015) used was obtained from the Resource and Environmental Science Data Centre (http://www.resdc.cn/).

## Data Records

In the dataset of distribution of antibiotic occurrence in solid environmental matrices in China (available from figshare^35^), each of the rows represents a single record (an occurrence of an antibiotic in a location as reported in a specific year by a reference). The 25 columns of this dataset are explained as follows:

1. sem_type: the type of solid environmental matrices (soil, sediments, or municipal sludge).
2. lon: the longitude of the location of antibiotic occurrence (WGS1984 Datum).
3. lat: the latitude of the location of antibiotic occurrence (WGS1984 Datum).
4. loc_level: the level of geographical details (1 = provincial level, 2 = prefectural level, 3 = county level, 4 = township or finer level).
5. loc_l1: provincial level information of the location (name of province, autonomous region, municipality, or special administrative region of China).
6. loc_l2: prefectural level information of the location (name of prefectural-level city, or autonomous prefecture).
7. loc_l3: county level information of the location (name of county-level city, autonomous banner, district, or county).
8. loc_l4: township or finer level information of the location.
9. loc_ref: supplemental geographical information of the location (river, lake, watershed, etc.).
10. ABX_cat: the categories of antibiotics (sulfonamides, tetracyclines, fluoroquinolones, macrolides, β-lactams, or other).
11. ABX_subcat: full name of antibiotic subcategories.
12. ABX_subcat_abbre: abbreviation of antibiotics subcategories.
13. ABX_subcat_CAS: the CAS (Chemical Abstracts Service) number of antibiotics subcategories.
14. smp_Y: the year of sampling.
15. smp_M: the month of sampling.
16. ABX_conc_max: maximum measured concentration of antibiotics (unit: μg/kg).
17. ABX_conc_min: minimum measured concentration of antibiotics (unit: μg/kg).
18. ABX_conc_mean: mean measured concentration of antibiotics (unit: μg/kg).
19. ABX_conc_median: median measured concentration of antibiotics (unit: μg/kg).
20. ABX_conc_mean_flag: if the mean measured concentration of antibiotics is a possible outlier, flag as 1, otherwise flag as 0.
21. ABX_dtm: abbreviation of analytical method employed (LC-MS/MS (Liquid chromatography-tandem mass spectrometry), HPLC-MS/MS (High performance liquid chromatography-tandem mass spectrometry), UPLC-MS/MS (Ultra performance liquid chromatography-tandem mass spectrometry), RRLC-MS/MS (Rapid Resolution Liquid Chromatography-tandem mass spectrometry), etc.).
22. pub_year: the year of the publication.
23. pub_id: identification number of references.
24. pub_full: references identified for data extraction.

## Technical Validation

There are 6929 records on the reported occurrence of antibiotics extracted from literature published between 2000 and 2020. All records were initially extracted by a review team. After the records were entered, a person checked the dataset thoroughly to ensure accuracy and avoid duplications. While at the stage of geo-positioning, another person was involved so that data were checked again. The whole team followed the same inclusion criteria to ensure the accuracy and validity of the occurrence records.

It is important to ensure that locations of antibiotic occurrence were duly georeferenced. This required intensive reading of the original publications and supplementary materials and analysing the semantics obtained from different sources. However, it was sometimes difficult to georeference the records due to an incomplete description of the sampling location. For example, some articles only mentioned unofficial names of places or objects in rural China (e.g. pond names in village) which had not been made identifiable from any online location services. Moreover, some articles only provided fine-level maps of sampling sites without any latitudinal and longitudinal grid, nor any geographical information which could enable visual interpretation. Furthermore, some studies focused on rivers or basins spanning across multiple administrative regions. It thus made it necessary to include the ‘loc_level’ and ‘loc_ref’ fields in the dataset, so that the readers are aware of our confidence in the spatial precision of each record.

The spatial distribution of antibiotic occurrence in solid environmental matrices in China was shown in Figs 3, 4, and 5. In general, studies on antibiotic occurrence in solid environmental matrices are mainly located in the coastal areas of China. For antibiotics in sediments, studies were clustered in the Liaohe River Basin (Liaoning and Shandong); the Haihe River Basin, surrounding Tianjin City; the Yangtze River Basin, particularly surrounding Jiangsu and Shanghai City; and the Pearl River basin, surrounding Guangdong. For antibiotics in soil, studies mainly clustered in the lower reaches of the Yangtze River and the Pearl River basins (Jiangsu, Zhejiang, and Guangdong). Most studies of antibiotics in sludge focused on Guangdong, Beijing, and Chongqing. All these regions are highly populated with intensive human activities.

The concentration levels of the six antibiotic subcategories varied with different solid environmental matrices (Table 1). The highest measured concentration of Fluoroquinolones was detected in sediments in the Yangtze River (Jiangsu section), with an average concentration of 44.27 μg/g^17^. The highest measured concentration of Macrolides was detected in municipal sludge in Wuxi, Jiangsu province, with an average concentration of 6890.95 μg/kg^39^. The highest measured concentration of Tetracyclines was detected in municipal sludge in Shijiazhuang, Hebei province, with an average concentration of 4063 μg/g^40^. The highest measured concentration of β-lactams was detected in sediments in the Ba River (Xi’an, Shaanxi province), with an average concentration of 43.00 μg/kg^41^. The highest measured concentration of Sulfonamides and Other were both detected in sediments in the Dagu Drainage River (Tianjin), with an average concentration of 4639.05 μg/kg and 5465.95 μg/kg, respectively^42^.

**Table 1:** Measured concentration (μg/kg) of six antibiotic subcategories in solid environmental matrices in China.

		FQs^b^	MLs^c^	TCs^d^	βLs^e^	SAs^f^	Other
Sediments	Max	44270.00	2669.53	234333.33	43.00	4639.05	5465.95
	Min	0.00	0.00	0.00	0.00	0.00	0.00
	Median	1.81	0.60	2.41	1.90	0.04	0.30
	IQR^a^	14.53	3.35	5.27	10.5	0.951	5.09
Soil	Max	651.60	3170.00	12900.00	5.26	2230.00	4600.00
	Min	0.00	0.00	0.00	0.58	0.00	0.00
	Median	3.88	0.29	9.60	2.79	0.11	0.20
	IQR	17.81	1.10	28.32	3.50	1.35	4.05
Municipal sludge	Max	11000.00	6890.95	4063000.00	10.35	2110.00	1401.50
	Min	0.00	0.00	0.00	0.00	0.00	0.00
	Median	154.47	13.40	306.47	0.00	4.90	4.10
	IQR	1401.50	60.30	1665.56	0.00	16.27	7.70

a IQR = interquartile range; b FQs = Fluoroquinolones; c MLs = Macrolides; d TCs = Tetracyclines; e βLs = β-lactams; f SAs = Sulfonamides.

Compared our dataset with the existing studies^2,6,20,21^, the resulting maps of antibiotic occurrence as depicted in Figs. 3–5 agree well with the previous findings^6,20,21^, except for the occurrence of antibiotics in soil. According to the research of Chen *et al*.^2^, there were no records of antibiotic occurrence in at least 15 provinces including Anhui, Henan, and Guangxi. However, in our dataset, the occurrence of antibiotics in soil has been widely reported, except for Hunan and four provinces in northwest China. Therefore, our study provides a more comprehensive picture of measured environmental concentrations of antibiotic occurrence in solid environmental matrices.

## Usage Notes

Our results show that antibiotics are ubiquitously presented in the solid environmental matrices in China. Being aware of the distribution of antibiotics is fundamental to support decision and direct actions to prevent and manage relevant pollutant emissions. The dataset described here could contribute to a more complete picture of the distribution of the reported antibiotic occurrences in the solid environmental matrices in China. The dataset is suitable to be used to investigate the spatio-temporal dynamics of antibiotic distribution at multiple scales. It can also be applied in the environmental and health risk assessment to identify potential sources of pollutants. The dataset has been designed so that potential users (environmental scientists, biotoxicologists, health geographers, policymakers, etc.) can easily filter or aggregate the dataset for their different investigation purposes.

It should be noted that the literature reviewed in this study adopted different methods for antibiotic identification and quantification, which may have introduced background uncertainty. For example, some early detection of antibiotics adopted the liquid chromatography-mass spectrometry (LC-MS)^43^ or the liquid chromatography fluorescence detection (LC-FLD)^44^. Most of the recent studies utilized the high performance liquid chromatography-tandem mass spectrometry (HPLC-MS/MS) system^11,18,45–48^, which is more efficient than only using the parent ions as in LC-MS analysis^36^.

## Supplementary information

Supplementary Information

## Data Availability

There is no custom code produced during the collection and validation of this dataset.
