# Supplementary material for: A dataset of distribution of antibiotic occurrence in solid environmental matrices in China
Source: Sci Data. 2022 Jun 7;9:276. doi: 10.1038/s41597-022-01384-5 (PMC9174198; doi:10.1038/s41597-022-01384-5)
Supplement: Supplementary file 1 — Supplementary Information [file 41597_2022_1384_MOESM1_ESM.pdf]

Supplementary Information

A dataset of distribution of antibiotic occurrence in solid environmental matrices in China

Qi Zhang[1], Guanshi Zhang[1], Dongsheng Liu[1], Xiu Zhang[1], Ruying Fang[1], Luqi Wang[1], Yunxiang Chen[1], Lingling Lin[1], Hongjuan Wu[1], Sen Li[1, 2]

1. School of Environmental Science and Engineering, Huazhong University of Science and Technology, Wuhan, P.R. China

2. Environmental Change Institute, University of Oxford, Oxford, UK

Corresponding author: Sen Li (senli@hust.edu.cn)

Contents

Supplementary Table 1. Relevant information and properties of antibiotics 1

Supplementary Table 1. Relevant information and properties of antibiotics

Class	Antibiotic	Abbreviation	CAS No.
	Sulfadimethazine	SDM	122-11-2
	Sulfisoxazole	SSZ	127-69-5
	Sulfadimoxine	SDX	2447-57-6
	Sulfaphenazole	SPZ	526-08-9
	Sulfamethazine	SMZ	57-68-1
	Sulfaquinoxaline	SQX	59-40-5
	Sulfanilamide	SNM	63-74-1
	Sulfameter	SME	651-06-9
	Sulfathiazole	STZ	72-14-0
	Sulfamethoxazole	SMX	723-46-6
	Ormetoprim	OMP	738-70-5
	Trimethoprim	TMP	6981-18-6
	Sulfachloropyridazine	SCP	80-32-0
	Sulfapyridne	SPD	144-83-2
Sulfonamides (SAs)	Acetyl sulfamethazine	ASMZ	100-90-3
	Sulfadimethoxine sodium salt	SSS	1037-50-9
	Sulfamonomethoxine	SMM	1220-83-3
	Sulfanitran	SNT	122-16-7
	Sulfabenzamide	SBZ	127-71-9
	Sulfamerazine	SMZ1	127-79-7
	Sulfacetamide	SCM	144-80-9
	Sulfamethizole	SMTZ	144-82-1
	Thiabendazole	TBZ	148-79-8
	Sulfadimethoxypyrimidine	SDT	155-91-9
	N4-Acetyl-Sulfamethoxazole	NSMX	21312-10-7
	Sulfisomidine	SSM	515-64-0
	Sulfaguanidine	SG	57-67-0
	Sulfadiazine	SDZ	68-35-9
	Sulfamoxole	SMO	729-99-7
	Sulfamethoxypyridazine	SMP	80-35-3
	Danofloxacin	DAN	112398-08-0
	Pefloxacin	PEF	70458-92-3
	Norfloxacin	NFX	70458-96-7
	Fleroxacin	FL	79660-72-3
Fluoroquinolones (FQs)	Ofloxacin	OFX	82419-36-1
	Ciprofloxacin	CFX	85721-33-1
	Enrofloxacin	EFX	93106-60-6
	Levofloxacin	LEV	100986-85-4
	Sparfloxacin	SPA	110871-86-8
	Gatifloxacin	GAT	112811-59-3

	Orbifloxacin	ORB	113617-63-3
	Marbofloxacin	MAR	115550-35-1
	Tosufloxacin tosylate	TSFX	115964-29-9
	Nadifloxacin	NDFX	124858-35-1
	Oxolinic acid	OLA	14698-29-4
	Cinoxacin	CIN	28657-80-9
	Moxifloxacin	MOX	354812-41-2
	Nalidixic acid	NDA	389-08-2
	Flumequine	FLU	42835-25-6
	Pipemidic acid	PIPA	51940-44-4
	Sarmoxicillin	SAMX	67337-44-4
	Enoxacin	ENO	74011-58-8
	Fluconazole	FCA	86386-73-4
	Sarafloxacin Hydrochloride	SRFX-HCL	91296-87-6
	Lomefloxacin	LFX	98079-51-7
	Lomefloxacin Hydrochloride	LOM-HCL	98079-52-8
	Sarafloxacin	SAR	98105-99-8
	Difloxacin	DIF	98106-17-3
Macrolides (MLs)	Kitasamycin	KIT	1392-21-8
	Josamycin	JSM	16846-24-5
	Dehydrated erythromycin	DETM	229318-98-3
	Erythromycin-H2O	ETM-H2O	23893-13-2
	Roxithromycin	RTM	80214-83-1
	Leucomycin	LCM	8025-81-8
	Tilmicosin	TIL	108050-54-0
	Erythromycin	ETM	114-07-8
	Tylosin	TYL	1401-69-0
	Clindamycin	CLIN	18323-44-9
	Clindamycin Hydrochloride	CLIN-HCL	21462-39-5
	Acetylspiramycin	ACE	24916-51-6
	Oleandomycin	ODM	3922-90-5
	Erythromycin A dihydrate	ETMA	59319-72-1
	Oleandomycin Phosphate	ODMP	7060-74-4
	Clarithromycin	CTM	81103-11-9
	Azithromycin	AZM	83905-01-5
Tetracyclines (TCs)	Doxycycline	DXC	564-25-0
	Tetracycline	TC	60-54-8
	Demeclocycline hydrochloride	DCTC-HCL	64-73-3
	4-Epichlortetracycline hydrochloride	EOTC-HCL	101342-45-4
	Doxycycline hydrochloride	DXC-HCL	10592-13-9
	Demeclocycline	DCTC	127-33-3
	Minocycline hydrochloride	MC-HCL	13614-98-7
	Doxycycline hyclate	DXC-HC	24390-14-5

	Methacycline hydrochloride	MTC-HCL	3963-45-9
	Metronidazole	MTZ	443-48-1
	Anhydro chlortetracycline	ACTC	4497-08-9
	Isochlortetracycline	ICTC	514-53-4
	Chlortetracycline	CTC	57-62-5
	Chlortetracycline Hydrochloride	CTC-HCL	64-72-2
	Tetracycline Hydrochloride	TC-HCL	64-75-5
	Oxytetracycline	OTC	79-57-2
	Methacycline	MT	914-00-1
	Epioxytetracycline	EOTC	14206-58-7
	α-apo-oxytetracycline	α-OTC	18695-01-7
	β-apo-oxytetracycline	β-OTC	18751-99-0
	Epianhydrotetracycline	EATC	4465-65-0
	Epitetracycline	ETC	79-85-6
β-lactams (βLs)	Cephalexin	CPX	15686-71-2
	Penicillin	PEN	69-57-8
	Penicillin V	PCV	87-08-1
	Penicillin V potassium salt	PCVP	132-98-9
	Cefazolin	CEZ	25953-19-9
	Amoxicillin	AMOX	26787-78-0
	Cefradine	CED	38821-53-3
	Cefmetazole	CFM	56796-20-4
	Penicillin G	PCG	61-33-6
	Cefotaxime	CFT	63527-52-6
	Cefotaxime Sodium	CTX	64485-93-4
	Ampicillin	AMP	69-53-4
	Ceftriaxone	CRO	73384-59-5
Other	Chloramphenicol	CAP	56-75-7
	Florfenicol	FF	73231-34-2
	Atenolol	ATE	29122-68-7
	Aureomycin	AM	64-72-2
	Lincomycin hydrochloride	LIN-HCL	859-18-7
	Minocycline	MH	10118-90-8
	Rifampicin	RIF	13292-46-1
	Gentamicin	GEN	1403-66-3
	Vancomycin	VAN	1404-90-6
	Thiamphenicol	TAP	15318-45-3
	Lincomycin	LIN	154-21-2
	Spectinomycin	SPE	1695-77-8
	Monensin	MON	17090-79-8
	Olaquindox	OQD	23696-28-8
	Mecillinam	MEL	32887-01-7
	Nicarbazin	NCZ	330-95-0
	Propranolol	PROP	5051-22-9

Salinomycin	SAL	53003-10-4
Narasin	NRS	55134-13-9
Streptomycin	STM	57-92-1
Cloxacillin	CLO	61-72-3
Cyromazine	CY	66215-27-8
Oxacillin	OX	66-79-5
Furazolidone	FZD	67-45-8
Metoprolol	MET	81024-43-3
